# Analysis and optimization of a Caco-2 cell culture model for infection with human norovirus

**DOI:** 10.1007/s00705-022-05437-3

**Published:** 2022-04-12

**Authors:** Clara Pohl, Grit Szczepankiewicz, Uwe Gerd Liebert

**Affiliations:** Department of Virology, University of Leipzig, Johannisallee 30, 04103 Leipzig, Germany

## Abstract

Human noroviruses (hNoVs) are an important cause of acute gastroenteritis worldwide. However, the lack of a reproducible *in vitro* cell culture system has impaired research and the development of preventive measures, therapeutic drugs, and vaccines. The aim of this study was to analyze and optimize a suitable cell line for *in vitro* cultivation of hNoV. The Caco-2 cell line, which is of colorectal origin and differentiates spontaneously into intestinal enterocyte-like cells, was chosen as a model. It was found that differentiated cells were more susceptible to infection with hNoV, resulting in a higher virus yield. This was accompanied by an increase in H type 1 antigen in the cell membrane during differentiation, which functions as an attachment factor for hNoV. Induced overexpression of H type 1 antigen in undifferentiated Caco-2 cells resulted in an increase in viral output to a level similar to that in differentiated cells. However, the relatively low level of viral output, which contrasts with what is observed *in vivo,* shows that the viral replication cycle is restricted in this model. The results indicate that there is a block at the level of viral release.

## Introduction

Human norovirus (hNoV) is the most common cause of non-bacterial gastroenteritis, with more than 684 million annual infections worldwide [22], and causes 18% of all acute gastroenteritis infections [2]. It was first discovered in 1968 during an epidemic outbreak at a primary school in Norwalk, Ohio, where 50% of the students and teachers were affected [1]. However, the lack of a reproducible cell culture system makes further characterization and understanding of the viral replication cycle and pathogenesis difficult and interferes with efforts to create a vaccine [25].

hNoV has a linear, non-segmented positive-sense RNA genome and belongs to the family *Caliciviridae*. The genome is enclosed in an icosahedral, non-enveloped capsid. Transmission occurs primarily via the fecal-oral route and is characterized by a high level of contagiousness [34]. Ten genogroups have been distinguished, three of which infect humans (genogroup I [GI], GII, and GIV). These are subdivided into almost 50 genotypes and their subtypes [6]. New variants emerge continually and cause epidemics especially during the winter months [2, 8].

Since its discovery, there have been multiple attempts to establish models for the cultivation of the virus under laboratory conditions. In the past years, there have been notable achievements, such as the establishment of a small-animal model using BALB/c Rag-γc-deficient mice [33] or a replication model for the hNoV genogroups GI and GII in zebrafish larvae [36]. In addition, two *in vitro* cell culture systems have been reported. However, both models achieved only modest levels of viral output, and passaging of the virus was limited [11, 19]. Thus, a better understanding of virus-host interactions is still necessary.

It remains unclear which steps of the viral replication cycle limit infection *in vitro*. Important insights have been gained using Huh-7 cells that were transfected with hNoV-RNA [13]. The transfection caused the cells to exhibit a clearly distinguishable cytopathic effect, and an increase in virus titer was measured in the supernatant. However, the viruses that were released were unable to infect adjacent cells. This observation led to the assumption that the replication cycle was blocked at early steps of infection, i.e., at the level of adhesion, penetration, or uncoating of the virus. These steps were bypassed by transfecting with the viral RNA directly, which led to successful infection of the cells [13].

The question has arisen as to which cellular receptor(s) and cofactor(s) might be used by hNoV to adhere to and penetrate cells. Candidate receptors that have been recognized to play a crucial role in hNoV infection are the histo-blood group antigens (HBGAs).

HBGAs are carbohydrate complexes on the surface of many cells, including erythrocytes, but also on the epithelium of the gastrointestinal and respiratory tract [28]. These carbohydrate complexes develop into the different blood group antigens by the stepwise addition of monosaccharides by fucosyltransferases (FUT1 and FUT2). FUT2 (or Se-enzyme) forms the precursors for blood group antigens that are secreted into saliva, breast milk, and the mucins of the gut as well as being attached to the epithelial cells of the gastrointestinal and respiratory tract [28]. The resulting trisaccharide is the so-called H antigen, which can be further modified to form the A, B, and Lewis blood group antigens.

Significant advancements have been made investigating the characteristics of hNoV-HBGA interaction. Volunteer challenge studies and outbreak investigations have shown a clear correlation between hNoV infection and HBGA phenotypes [18, 23]. Specific interactions between the diverse hNoVs and polymorphic HBGAs were observed using *in vitro* assays [24],reviewed in [17], and X-ray crystallography has been used for the analysis of the structural basis of hNoV-HBGA interactions [reviewed in [32]. The results suggest that tissue-bound HBGAs function as attachment factors for hNoV. More specifically, the results of immunohistochemical analysis, ELISA assays, and experiments measuring biomolecular interactions have suggested that hNoV-virus-like particles (VLPs) bind most tightly to H type 1 trisaccharides, whereas binding to H type 2 trisaccharides was found to be weaker [17, 31]. However, various studies investigating the subtype-dependent binding specificity and the mechanism of adhesion showed a large amount of variation in the ability of hNoVs to recognize polymorphic human HBGAs [reviewed in [31, 32]. An example is genotype GII.4, which has the highest prevalence worldwide due to a high rate of mutation and broad specificity for cellular receptors [31, 32].

The aim of this study was to identify a suitable cell line and to establish an *in vitro* cell culture model for hNoV. Caco-2 cells, which are of colorectal origin, proved most suitable based on previous findings using long-term cell culture [27] and considering the characteristics of hNoV infection *in vivo*. Upon differentiation, the cells functionally and morphologically adopt characteristic features of enterocytes within 14 to 21 days after reaching confluence [29]. This is of special interest, since biopsy studies evaluating the infection of gut tissue *in vivo* have found that norovirus infection targets the human small intestine [9, 30]. Furthermore, investigations using multicellular human intestinal enteroids derived from stem cells of duodenal, jejunal, and ileal tissue have shown replication in all three segments, but only enterocytes were infected [11]. Although hNoV was reportedly not able to establish a productive infection in Caco-2 cells, binding of the virus to the cells was shown by immunofluorescence assays [10]. In this study, we focused on the effect of differentiation of long-term cultivated Caco-2 cells on hNoV replication.

## Materials and methods

### Intestinal cell cultures

Three intestinal cell lines as well as the cell line HEK 293T were used for investigations. The Caco-2 cell line was kindly provided by Prof. Birchmeier (Max Delbrueck-Zentrum, Berlin, Germany) and applied at their ninth passage. The DLD-1 and HuTu-80-cell lines were purchased from Cell Lines Service GmbH (Eppelheim, Germany), and HEK 293T cells were acquired from the American Type Culture Collection (Manassas, VA, USA). Caco-2 and DLD-1 cells originated from colorectal adenocarcinomas, and the HuTu-80 cell line is of duodenal origin [10, 35]. The cells were cultivated at 37°C, 5% CO_2,_ and 95% humidity. All formed adherent monolayers in 25- or 75-cm^2^ cell culture flasks and were subcultivated at 80-90% confluence with a split ratio of 1:5 to 1:10. Caco-2 and HEK 293T cells were maintained in Dulbecco's modified Eagle's minimum essential medium (DMEM) with Glutamax I, whereas DLD-1 and HuTu-80 cells were cultured in DMEM F-12. The medium was supplemented with antibiotics (100 U of penicillin and 100 µg of streptomycin per ml), 10% fetal bovine serum (FBS), and for DLD-1 and HuTu-80 cells, additionally with 2 mM glutamine. All reagents were obtained from Thermo Fisher Scientific, San Diego, USA.

The cells were tested regularly for mycoplasma contamination by staining with bisbenzimide (Sigma-Aldrich, Steinheim, Germany) and by mycoplasma DNA-specific PCR.

### Caco-2 cell line: differentiation assay

The growth properties of all cell lines were determined in duplicate. Two flasks were trypsinized daily, and the cells were counted. The formation of liquid-filled elevations (so-called domes) within the cell monolayer of Caco-2 cells was documented daily using a Leica DM IL LED inverse light microscope (Leica Microsystems, Wetzlar, Germany).

A *p*-nitrophenol phosphate (pNPP) Tablet Kit (Sigma-Aldrich, Steinheim, Germany) was used to quantify the activity of alkaline phosphatase. The activity of the enzyme increases when confluence of the cellular monolayer is reached and serves as a marker for the differentiation of Caco-2 cells [12]. Its activity was measured following the producer's instructions. The hydrolysis of the pNPP by cellular alkaline phosphatase led to the release of *p*-nitrophenol, which has a yellow color and was quantified by measuring absorbance at 405 nm. The amount of *p*-nitrophenol produced is proportional to the change in absorbance and to the activity of the alkaline phosphatase in the lysate. To establish a standard curve, serial dilutions of alkaline phosphatase (Roche Diagnostics, Mannheim, Germany) were performed.

Immunofluorescent staining was used to visualize the formation of tight junctions between cells and the presence of the H type 1-antigen on the monolayer surface. The antibodies used for these stainings were obtained from Thermo Fischer Scientific (San Diego) (ZO-1 monoclonal antibody [ZO1-1A12] labeled with Alexa 488; human blood group antigen H1 antibody (17-206) labeled with Alexa 488). Tetramethylrhodamine isothiocyanate (TRITC)-linked lectin *Ulex europaeus* agglutinin I (UEA-I) was purchased from Biozol (Eching, Germany) and used to evaluate the expression of H type 2 antigen on Caco-2 cells. A Leica DM1000 LED microscope was used for imaging.

### Infection experiments

For infection experiments, 11 hNoV-positive fecal samples (10 genogroup GII.4; 1 GI.3) that were left over from the material collected during the season 2016/2017 were used. The samples were stored at -20°C until used in infection assays. The unfiltered stool samples were diluted 1:10 in phosphate-buffered saline (PBS) and used to infect confluent cell monolayers in 24-well plates. A 200-µl aliquot of the stool suspension used for the infection was put aside to determine the number of viral genome copies in the inoculum. In six triplicate experiments, the virus inoculum was removed, the cell cultures were washed, and the NoV genome copy number was found to be below 5 × 10^3^. In the infection experiments, 11 fecal samples were used to infect cells at four differentiation time points (6 , 13, 20, and 27 days after plating) in parallel. For each pair of a stool samples with a cellular differentiation state, the cells in two wells were infected, and lysates and supernatants were collected on days 1 and 5 after infection, resulting in 88 parallel infection assays. Also, a mock infection was performed simultaneously, using an hNoV-negative stool sample. An infection of Caco-2 cells at 62 days after plating was performed separately, but under identical conditions, using the same aliquoted 11 hNoV-positive stool samples and mock infection sample. Supernatant samples and cell lysates were collected on days 1 and 5 after infection. Other assays were conducted in order to assess the effect of variables such as the gentle centrifugation of the infected monolayer at 1000 rpm for 5 min and the addition of 2% FBS to the supernatant one day after infection to enhance cell vitality. Infection of FUT2-overexpressing cells was performed using four different stool samples that tested positive for hNoV GII.4. Virus was passaged with cell lysate as well as intact infected Caco-2 cells in co-culture. For this purpose, infected cells were washed and trypsinized 4-6 days after the primary infection. Each sample was passaged in confluent Caco-2 cells and, in parallel, as lysate and as co-culture. To lyse the infected cells, they were frozen at -80°C and thawed three times. Additionally, DLD-1 and HuTu-80 cells were infected.

Nucleic acids were extracted from the supernatant and the cellular lysate at 3-4 hours and 5 days after infection (MagNA Pure 96 DNA and Viral NA Small Volume Kit, Roche, Germany) and reverse transcription (GeneTouch Thermal Cycler, Biozym, Germany), followed by a real-time PCR (LightCycler 2.0, Roche, Germany) with the probes NoroITaqYAK and NoroIITaqFAM (both purchased from TibMolBiol, Germany), was used to determine the amount of hNoV RNA in the sample. The target sequence was the ORF-1/ORF-2 junction between the polymerase and capsid genes [21]. The primers used for this purpose were as follows: For genogroup GI, the forward and reverse primers were 5′-GCYATGTTCCGCTGGATGC-3′ and 5′ CGTCCTTAGACGCCATCATCA-3′, respectively [15]. For genogroup GII, the forward and reverse primers were 5′-CARGARBCNATGTTYAGRTGGATGAG-3′ and 5′-TCGACGCCATCTTCATTCACA-3′, respectively [21]. All primers were purchased from Metabion, Germany.

Immunofluorescent staining of infected Caco-2 cells was performed using a monoclonal antibody against the viral G2 epitope on the hNoV capsid (catalog number: 10-2735; Fitzgerald Industries International, Acton, USA) and a secondary Alexa Fluor 488–linked goat anti-mouse antibody (Invitrogen GmbH, Karlsruhe, Germany). This was repeated three times in undifferentiated (2 days after plating) as well as long-term-cultured (20 days after plating) Caco-2 cells, and a mock infection using UV-inactivated (8W, 15 s) hNoV-positive stool samples was performed in parallel.

### Transfection

For transfection and overexpression of the FUT2 enzyme in Caco-2 cells, the eukaryotic expression vector pCMV6-FUT2-GFP was used (OriGene Technologies, Rockville, USA). This plasmid contains a CMV promoter. The gene for α-1,2-fucosyltransferase 2 is inserted into the multiple cloning site (MCS) together with the gene for the green fluorescent protein (GFP), which can be detected by immunofluorescence or flow cytometric analysis after transfection. This insert is followed by a polyadenylation signal. For bacterial transformation, the vector possesses a pColE1 origin of replication (ori) and a gene coding for resistance to ampicillin.

The plasmid was propagated by transformation of chemically competent *E. coli* bacteria (NEB-5, New England Biolabs, Ipswich, USA). The presence of the correct insert was verified by colony PCR and plasmid PCR as well as by sequencing of the entire FUT2 insert and by digestion with the restriction enzymes EcoRI-HF (New England Biolabs, Ipswich, USA)*,* NotI, and BtsEII (Thermo Fisher Scientific, San Diego, USA). Plasmid isolation was performed following a ZymoPURE ™ Plasmid MaxiPrep Kit (Zymo Research, Freiburg i. Br., Germany) according to the manufacturer’s recommendations.

For transfection assays, Lipofectamine 2000 (Invitrogen, Karlsruhe, Germany) was used as a biological transfection reagent. Caco-2 cells were transfected in order to overexpress the FUT2 gene. HEK 293T cells were transfected in parallel as a transfection control. The success of transfection was assessed by immunofluorescent and flow cytometric visualization of GFP expression as well as by labeling the H antigens with a TRITC-linked lectin, UEA-I, and performing immunofluorescent imaging. The UEA-I lectin binds to Fuc-α-1,2-Gal-epitopes on fucosylated oligosaccharides and therefore especially to H type 1 and H type 2 antigens.

### Flow cytometry

A BD Accuri C6 flow cytometer (BD Biosciences, San Jose, USA) was used to analyze cell properties and fluorescent staining. The cells were either fixed in 2% paraformaldehyde for 15 min or analyzed without fixation. Between washing steps, the cell suspension was centrifuged at 1200 rpm for 4 min at 4°C to separate the supernatant from the cells. Cells were also stained with an Alexa–488-linked H1 antibody or a TRITC-linked UEA-I lectin. This staining was performed in a single-cell suspension in PBS with 1% BSA after fixation with 2% paraformaldehyde. The analysis was performed on the FL1 channel of the flow cytometer for GFP- and Alexa 488–labelled substrates and on the FL2 channel for the TRITC-labeled UEA-I.

## Results

### Characterization of Caco-2 cells

**Fig. 1:**
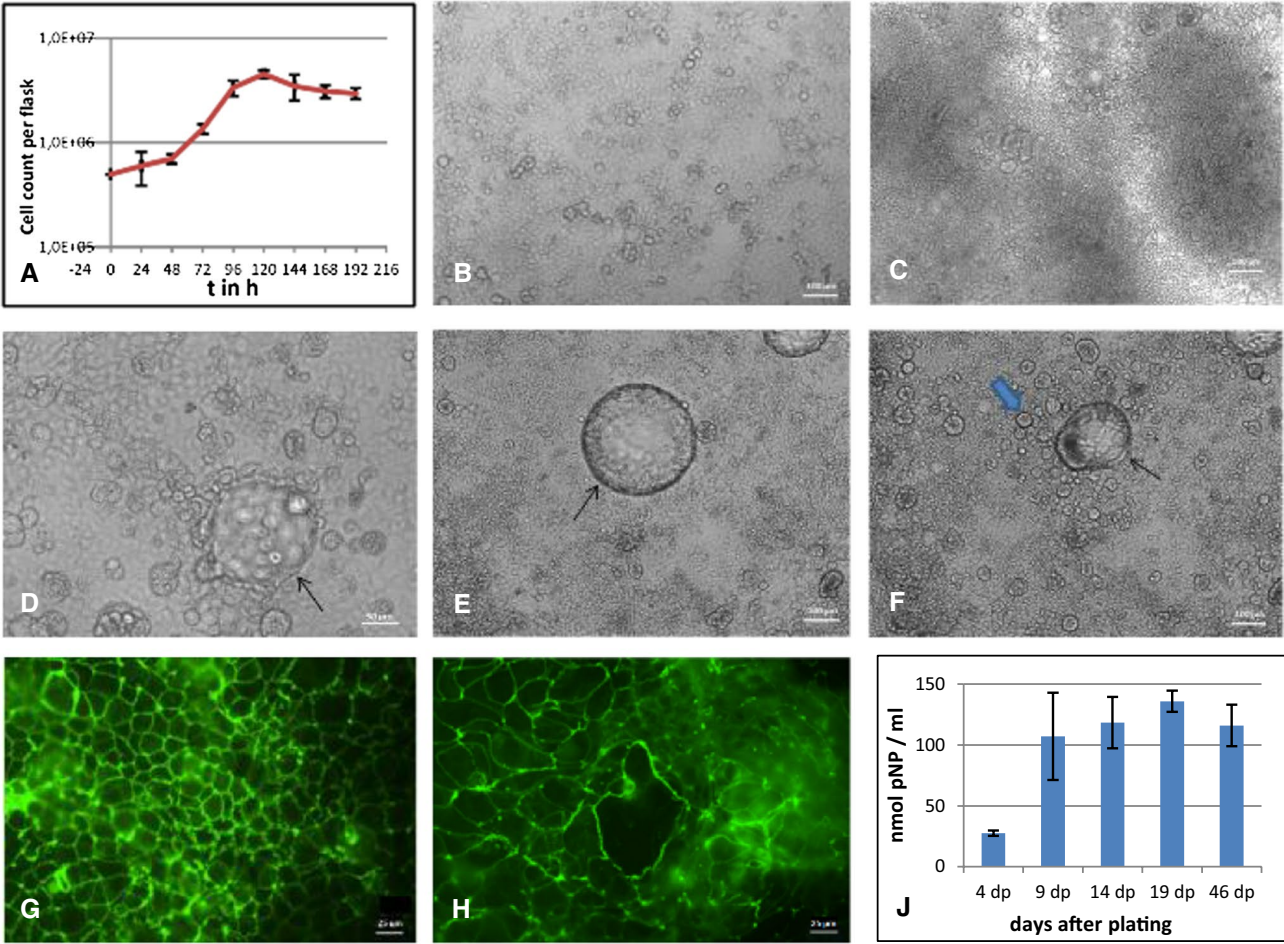
Characteristic features of Caco-2 cell monolayers during differentiation. (A) Growth curve of Caco-2 cells. The values presented are the mean of two determinations. The vertical bars represent standard deviation. (B and C) Light microscopic images of Caco-2 cells on day 4 (B) and day 27 (C) after plating (dp). (D-F) Light microscopic images of domes in the monolayer of Caco-2 cells. Domes are indicated by thin arrows. They protrude from the monolayer into a different focus level (D, day 7), initially increase in diameter (E, day 18), and eventually either burst, leaving a hole in the epithelial layer, or collapse, releasing floating cell lumps (F, day 19). A thick blue arrow points to a cell lump. (G and H) Immunofluorescent imaging of the tight junctions in a Caco-2 cell monolayer at day 5 (G) and day 21 (H). Caco-2 cells were stained with a monoclonal antibody against zonula occludens protein 1. (J) Enzyme activity of the intestinal alkaline phosphatase during differentiation of Caco-2 cells, measured using a *p*-nitrophenol phosphate assay. Caco-2 cells were cultivated for the indicated time periods. The amount of *p*-nitrophenol produced is proportional to the change in absorbance and to the activity of the alkaline phosphatase in the lysate. The values presented are the mean of four determinations; the vertical bars represent the standard deviation.

Caco-2 cells showed a plateau of proliferation about four days after plating (Fig. 1A). These results match the observations that were made with light microscopic imaging: Caco-2 cells were confluent after being held in culture for four days. The process of growth and differentiation of the Caco-2 cells was monitored and documented by daily light microscopic imaging for a period of two months (Fig. 1B-F). The epithelial cells grew increasingly more inhomogeneous. In some spots, the monolayer became very dense and three-dimensional, forming so-called domes. These elevations correspond to differentiation into transporting epithelia, and the cells retain their potency to polarize *in vitro* and express directed salt and water transport and intact cell-cell interactions, forming a water-impermeable layer [14]. Other areas remained a flat monolayer. The formation, collapse, and burst of domes was also documented with light microscopic imaging (Fig. 1D-F).

Morphological characteristics of small-intestine enterocytes, such as the formation of intercellular tight junctions were visualized on days 5 and 21 after plating, using a monoclonal antibody against zonula occludens protein 1 (Fig. 1G and H), and showed highly developed intercellular contacts in the confluent monolayer. Differentiation of the cellular monolayer over time was indicated by an increase in intestinal alkaline phosphatase activity in the cell culture supernatant, which was measured at various time points after plating. A clear increase in intestinal alkaline phosphatase activity was measured between days 4 and 9, with a plateau observed after two weeks of cultivation (Fig. 1J).

**Fig. 2:**
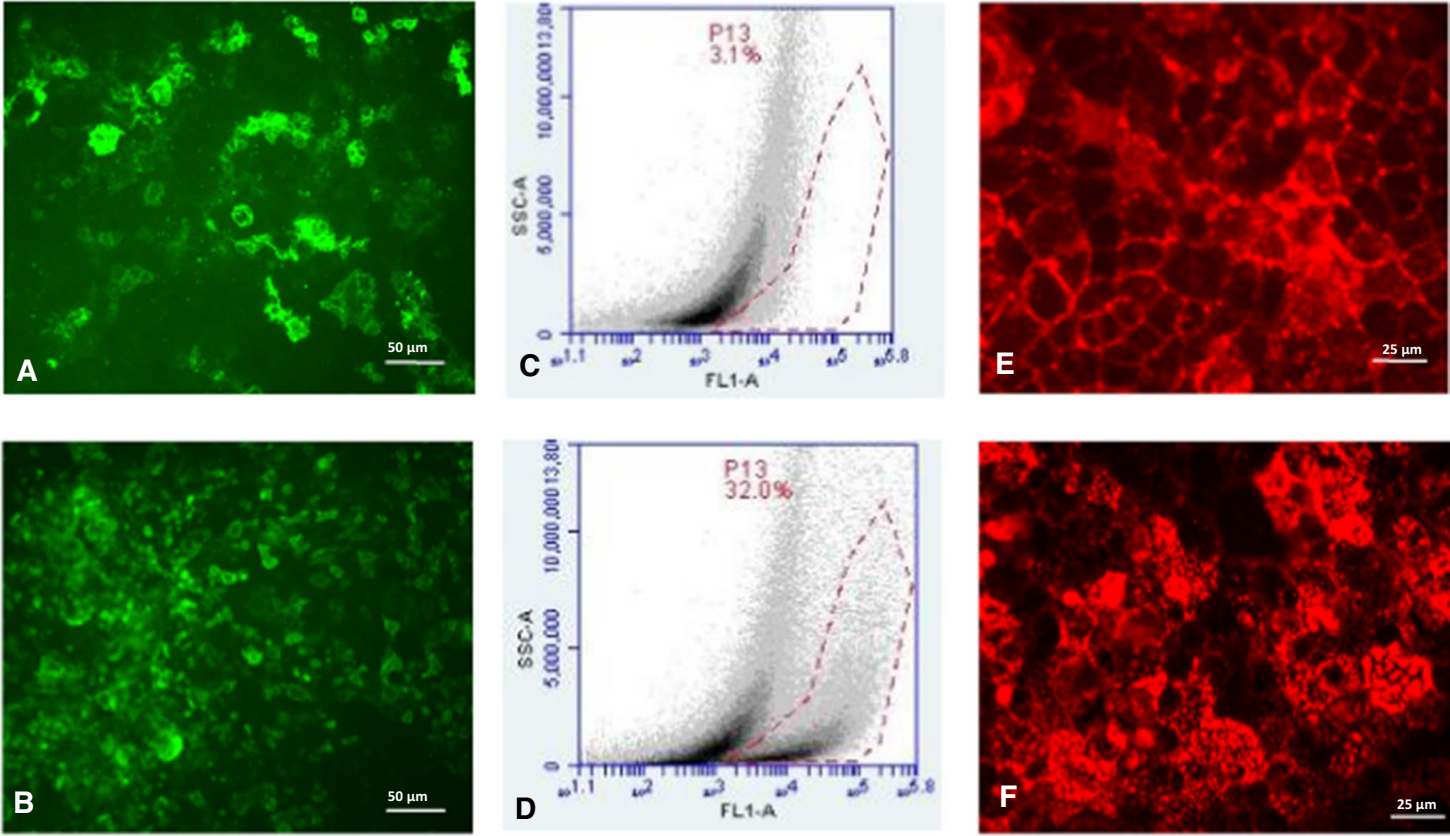
Expression of H type 1 and H type 2 antigens on Caco-2 cells. (A and B) Fluorescent staining of Caco-2 cells with a monoclonal antibody for the H type 1 antigen (labelled directly with Alexa 488) on day 4 (A) and day 27 (B) after plating. (C and D) Representative images of Caco-2 cells stained with a monoclonal antibody recognizing the H type 1 antigen (labelled with Alexa 488) and subjected to flow cytometric analysis. The cells shown here were kept in culture for 3 (C) or 60 (D) days before analysis. The area within the dotted red line contains cells showing higher fluorescence intensity. (E) Fluorescent staining of undifferentiated Caco-2 cells at day 4 after plating with the TRITC-linked UEA-I. (F) Fluorescent staining of Caco-2 cells at day 27 after plating with the TRITC-linked UEA-I*.*

Immunofluorescent staining was performed using a monoclonal antibody for the H type 1 antigen as well as the UEA-I lectin. Parallel staining of differentiated and undifferentiated Caco-2 cells showed an inhomogeneous pattern of expression of both antigens (Fig. 2). The percentage of cells expressing the H type 1 antigen appeared to be higher in the monolayer of differentiated cells (Fig. 2A and B). These results were confirmed using flow cytometry. Native Caco-2 cells were stained and examined before and after reaching confluence, and they showed characteristics of differentiation such as the formation of domes. While undifferentiated cells homogeneously showed weak fluorescent staining by the H type 1 antibody (directly Alexa 488 labelled), the differentiated cells could be subdivided into two populations: 68% showed weak staining similar to the undifferentiated cells, while 32% showed a 7.5-fold increase in fluorescence, suggesting increased H type 1 antigen expression (Fig. 2C and D).

The expression of H type 2 antigen was examined using differentiated and undifferentiated Caco-2 cells stained with the TRITC-linked UEA-I lectin and comparing these to cells stained with the H type 1 antibody, which had been plated in parallel. Immunofluorescence and flow cytometry showed no difference in H type 2 expression between the two cell populations (Fig. 2E and F). Differences in appearance were considered to be due to an increase in inhomogeneity of the Caco-2 monolayer over time.

### Infection experiments with native Caco-2 cells

**Fig. 3:**
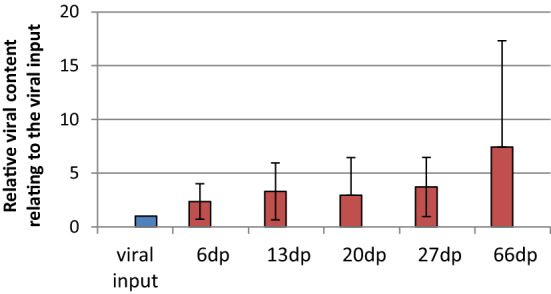
Relative number of viral genome copies in cell lysates 5 days after infection. Caco-2 cells were plated at different time intervals and infected in parallel. After an incubation period of five days, the cells were lysed and the amount of norovirus RNA in the lysate was determined by quantitative reverse transcription real-time PCR. The amplification level was calculated as the ratio of viral content to viral input. The mean input number of viral copies in this assay was 6.08 × 10^8^. The values presented are the mean of six measurements; the vertical bars represent the standard deviation.

Based on the observation that differentiated Caco-2 cells express larger amounts of H type 1 antigen, we investigated whether this leads to higher virus output in differentiated cells infected with hNoV. Cells were infected in parallel at five different time points (6, 13, 20, 27, and 66 days after plating), and the mean relative number of viral copies compared to the virus input was determined (Fig. 3).

An increase of up to seven-fold in the mean virus output was observed in differentiated cells However, a single-factor analysis of variance showed no significance.

**Fig. 4:**
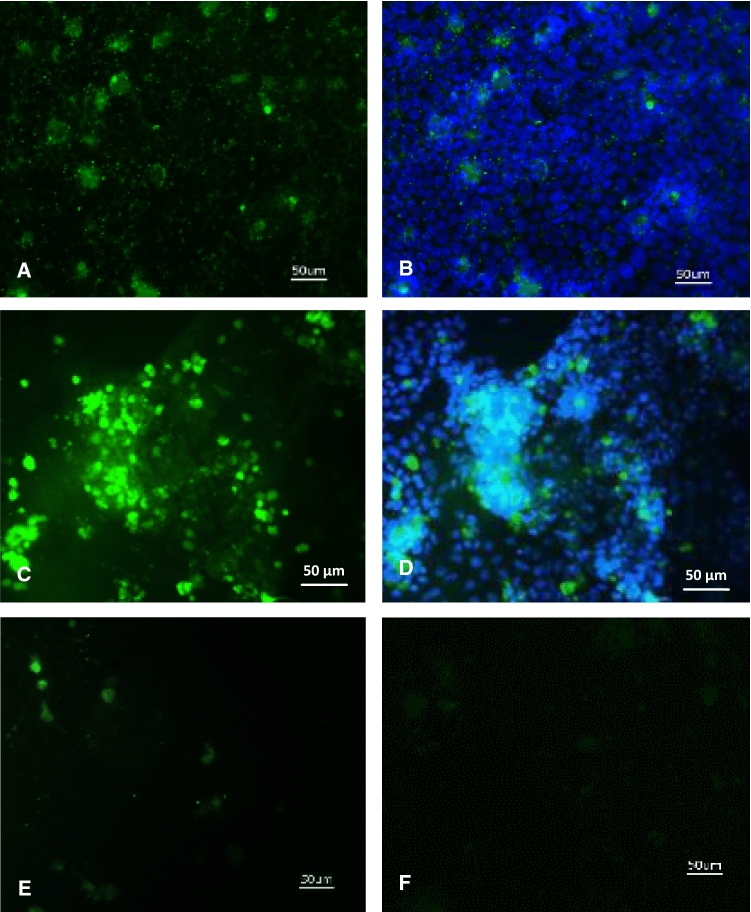
Immunofluorescence detection of hNoV antigen in infected undifferentiated (A and B) and infected differentiated (C and D) Caco-2 cells. Cells that were mock infected with a norovirus-negative stool sample (E) and infected cells stained with the secondary antibody only (F) were used as controls. Undifferentiated (day 2 after plating) and differentiated Caco-2 cells (day 20 after plating) were infected with an hNoV-positive stool sample in parallel. In both cases, 1.33 × 10^7^ viral copies were used for inoculation. Staining with an hNoV G2 antibody and a secondary Alexa Fluor 488 linked goat anti-mouse antibody was performed two days after infection. The successfully infected cells show green staining. Overlay images of hNoV staining with DAPI-staining show cell nuclei (B and D).

Comparative immunofluorescent staining of undifferentiated and differentiated Caco-2 cells infected with norovirus-positive fecal samples was performed using a monoclonal antibody recognizing the norovirus G2 epitope. Caco-2 cells were infected at day 2 or day 20 after plating. Higher fluorescence intensity was seen in differentiated cells, and it was observed that the fluorescent cells grew in clusters that correlated to areas of dense cellular growth within the epithelial monolayer (Fig. 4).

**Fig. 5:**
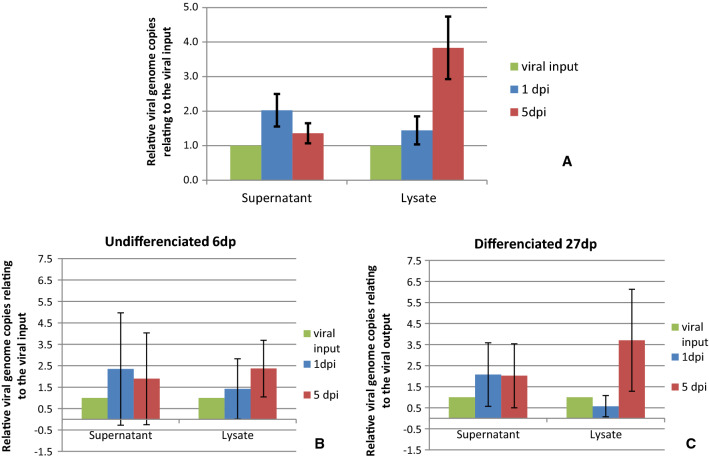
Mean relative number of viral genome copies in the supernatant and lysate of infected Caco-2 cells at days 1 and 5 after infection as determined by PCR, independent of their differentiation time point (A), at 6 days after plating (B), and at 27 days after plating (C). Various samples with different virus concentrations ranging from 8.4 × 10^5^ to 4.68 × 10^9^ virus copies per infected well were applied. The amplification level was calculated as the ratio to viral input. The values presented in panel A are the mean of 29 determinations for the supernatant on day 1 and 5 days postinfection (dpi), respectively, and of 21 determinations for the lysate. The values presented in panels B and C show the mean of six determinations; the vertical bars represent the standard deviation.

While the mean number of viral copies in the supernatant decreased from day 1 to day 5 after infection, it increased in the lysate in all samples (Fig. 5). A one-sided pairwise comparison with dependent samples was performed in order to compare the relative viral content on day 5 after infection in supernatants and lysates. The *p*-value (*p* = 0.0049) showed a highly significant difference, with more virus copies found in the lysate than in the supernatant (Fig. 5A). This tendency was more pronounced in differentiated Caco-2 cells 27 days after plating than in undifferentiated cells. The mean viral content in the cell lysate of the differentiated cell population increased more than six-fold (Fig. 5B and C). Altered infection conditions such as gentle centrifugation (1000 rpm for 5 min) or the addition of 2% fetal bovine serum on day 1 postinfection did not result in increased viral output. Also, passaging of the virus was not successful, as no viral genome copies were detected after more than two passages using the lysate of infected cells as well as intact infected cells in co-culture.

Infection experiments with other intestinal cell lines (DLD-1 and HuTu-80 cells) also yielded no indication of viral replication.

### Transfection assays

In preparation for transfection assays, competent *E. coli* bacteria were transformed with the pCMV6-FUT2-GFP vector, and the insert was confirmed by colony PCR, restriction digestion, and sequencing (data not shown). At the protein level, H antigen expression was detected using immunofluorescent imaging and flow cytometry. In addition to the pCMV6-FUT2-GFP-transfected cells, native and pmiRneg*-*transfected Caco-2 cells were tested in parallel, and HEK 293T cells were transfected with the pCMV6-FUT2-GFP plasmid as a control.

**Fig. 6:**
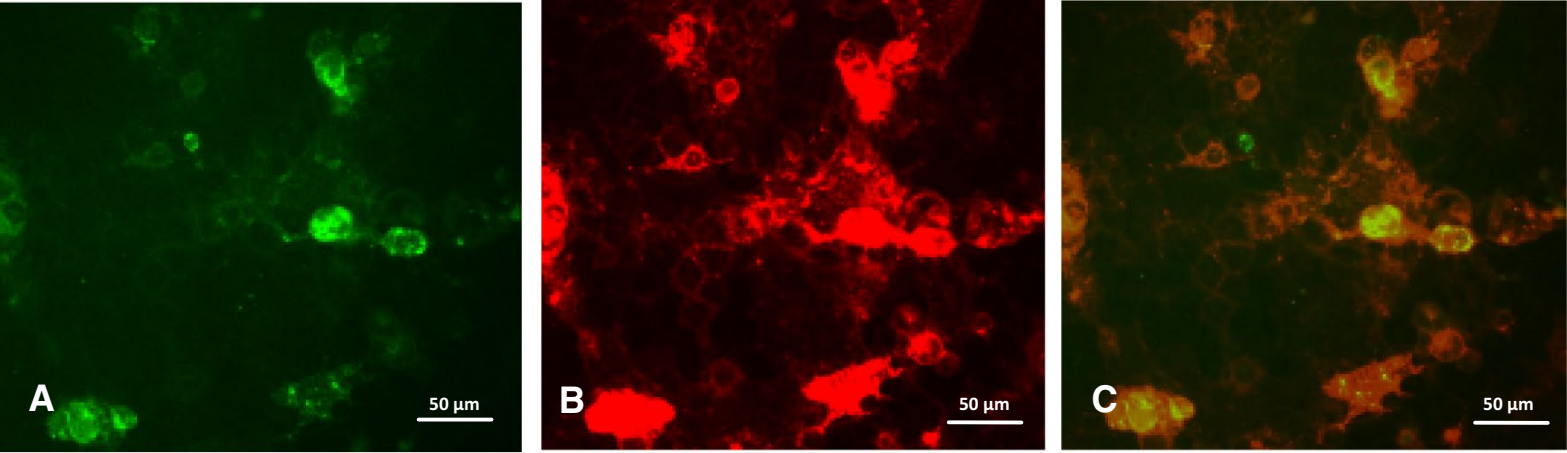
Transfection efficiency and detection of HBGA overexpression on pCMV6-FUT2-GFP transfected Caco-2 cells. Cells were transfected with the pCMV6-FUT2-GFP plasmid, blocked, and stained with a TRITC-conjugated UEA-I lectin 48 h after transfection. (A) Green fluorescence of transfected cells expressing GFP. (B) Red fluorescence of Caco-2 cells stained with UEA-I lectin, which indicates H type 1 and H type 2 antigen expression. (C) Overlay of plasmid expression and HBGA detection. Cells with simultaneous green and red fluorescence overexpress the H type 1 antigen.

The Caco-2 cells showed a transfection efficiency of less than 10%, whereas control HEK 293T cells had a transfection efficiency of 70 to 80% based on cell counting in immunofluorescence images (not shown). The cells were stained with the lectin UEA-I, and an overlay analysis showed that the transfected cells, which exhibited green fluorescence due to the GFP sequence included in the transfecting plasmid, expressed more H antigen on their surface than did the native Caco-2 cells (Fig. 6). Native cells that did not show green fluorescence also exhibited a lower intensity of red fluorescence after staining with TRITC-linked UEA-I.

Flow cytometric analysis of transfected cells stained with UEA-I reproducibly showed that approximately 12% of the examined cells showed a higher level of fluorescence than native Caco-2 cells (data not shown).

### Infection experiments with FUT2-overexpressing Caco-2 cells

**Fig. 7:**
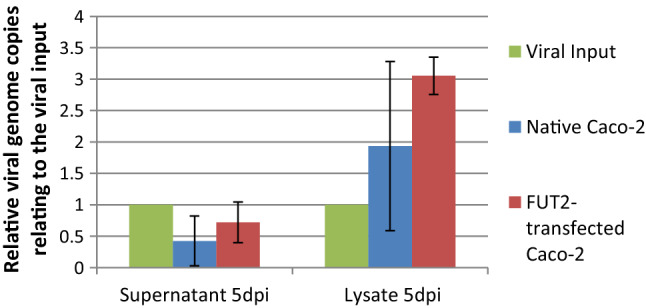
Comparison of the mean relative number of viral genome copies in native and FUT2-overexpressing Caco-2 cells infected with hNoV. Native as well as pCMV6-FUT2-GFP-plasmid-transfected Caco-2 cells were infected in parallel with hNoV-positive fecal samples. The amount of norovirus RNA was measured using a quantitative reverse transcription real-time PCR, and the mean relative number of viral genome copies was calculated in relation to the viral input. The values presented are the mean of three determinations; the vertical bars represent the standard deviation.

Native and FUT2-transfected cells were infected with three hNoV-positive fecal samples in parallel under identical conditions. This assay was repeated three times, and the mean viral output was calculated. A correlation was seen, showing a higher virus output in FUT2-transfected cells, especially in the lysate, at day 5 after infection (Fig. 7). In the lysate, the mean number of viral genome copies was higher in the FUT2-overexpressing cells. It more than tripled in the transfected cells. However, due to the small sample size, no significant difference in the viral replication rate could be observed.

## Discussion

In this study, we found that differentiated Caco-2 cells provide higher viral output than undifferentiated cells when infected with hNoV-positive stool samples. We show that this correlates with higher expression of HBGA, and specifically the H type 1 antigen, on differentiated cells. Undifferentiated Caco-2 cells were infected with similar productivity when overexpression of the H type 1 antigen was induced. We also found that the blockage of viral replication most likely occurred at the level of viral release.

It was shown in this study that differentiated Caco-2 cells are susceptible to infection with hNoV, although the viral output was low, and passaging of the virus was not possible. This contrasts with previous studies using short-term Caco-2 cell cultures, where no productive infection with hNoV could be established [10]. It was shown previously that Caco-2 cells differentiate morphologically and biochemically to resemble mature enterocytes [37]. During this process of differentiation, the expression of H type 1 antigen increases, whereas H type 2 antigen expression remains essentially unaltered, in accordance with published results [4]. The data presented here confirm these findings. This is of interest, since HBGAs, and especially H type 1 antigen, have been identified as attachment factors for infection with this virus [31, 32]. Caco-2 cells were infected with hNoV-positive stool samples at various time points after plating, and a correlation of cell age with their susceptibility to hNoV was seen.

In order to assess if this was due to a higher level of H type 1 antigen expression on differentiated cells, a comparative infection of FUT2-transfected, H-type-1-antigen-overexpressing cells and native Caco-2 cells was done. The results indicate a positive correlation of H type 1 overexpression with higher virus output after infection. The virus output of undifferentiated, H-type-1-antigen-overexpressing cells was comparable to that of differentiated Caco-2 cells. It is therefore conceivable that the increased susceptibility of differentiated Caco-2 cells to hNoV infection may be, among other yet undefined factors, due to their higher expression of H type 1-antigen.

Another important observation was made when comparing the virus content in the supernatants of Caco-2 cell cultures and cell lysates. Mean viral copies in the supernatants decreased from day 1 to day 5 after infection, but in the lysates, they increased during the same period. This was more marked in differentiated than in undifferentiated Caco-2 cells. These results could indicate that the replication cycle of hNoV is restricted at the level of viral release rather than during penetration and replication of the virus. In this case, the virus would be able to penetrate the cells, resulting in a decrease in viral content in the supernatant. Replication would lead to an increase in viral copies in the cells and therefore in the lysate. However, due to a blockage of the release of virions from the infected cells, a productive infection would not be possible. This is supported by the lack of a cytopathic effect (CPE) in infection experiments, but it is in contrast to the observations and conclusions made by Guix et al., who found that the viral replication cycle is blocked at early steps, i.e., penetration or uncoating, when using hepatocellular-carcinoma-derived Huh-7 cells *in vitro*, and who also reported a clearly distinguishable CPE after these cells were transfected with hNoV-RNA [13]. These observations could therefore possibly be cell-type-related due to the use of Huh-7 rather than intestinal cells. This may also explain the unsuccessful attempts to establish a productive cell culture system for hNoV based on Caco-2 cells, although binding of the virus to this cell line was reproducibly detected by immunofluorescence assays [10]. A step to further assess this observation could be an extended passage study of infected Caco-2 cells, including a lysis step before passaging.

Apart from the Caco-2 cells presented here, two *in vitro* culture systems are currently available for hNoV, although neither produces the high viral titers that are typically achieved with other cultivable caliciviruses *in vitro*. The first published model was a B cell line (BJAB) in co-culture with intestinal bacteria [20]. B cells were considered possible targets based on observations that had been made with murine NoV. The second model consists of human intestinal enteroids (HIEs), a more complex, non-transformed replica of the intestinal epithelium, consisting of enterocytes, enteroendocrine cells, goblet cells, and Paneth cells [7, 11]. Although further optimization and investigation of these established models might be the most logical and direct path to a productive *in vitro* cell culture system for hNoV, other means may also lead to a better understanding of virus pathology and replication, and other factors besides cell tropism must be considered.

For example, it will be crucial to further our understanding of the mechanisms of adhesion and penetration of the virus. Guix et al. showed that overexpression of HBGAs resulted in increased binding of hNoV virus-like particles. However, these particles were not internalized and did not lead to an infection [13]. Also, it is known that several hNoV subtypes bind completely independently of HBGAs [16]. Some capsids of such subtypes have been reported to bind to and become internalized in the intestinal epithelium of ileal biopsies [26]. Therefore, additional receptors and cofactors might be important. Although the mechanisms of interaction of hNoV with these molecules are still mostly unknown, a few hypotheses can be made, as particular chemical, biological, and bacterial components have been identified [reviewed by [3]. Other observations pointed to the conclusion that epithelial cells are not the only biological target of hNoV infection, but immune cells are additional hosts under certain conditions [reviewed in [5, 38].

### Limitations

This study is a proof-of-principle examination of the suitability of *in vitro*-differentiated Caco-2 cells as a cell culture system for hNoV. Due to the high diversity of hNoV genotypes and their subtypes, further efforts with a larger sample size and other genotypes are necessary. The relatively low level of virus output in cell culture is in contrast to the fulminant clinical presentation of hNoV infection, characterized by the shedding of a very large numbers of virus particles in the patient’s stool. This could be explained by the factors discussed, the inhomogeneity of the Caco-2 cell monolayer, or a block of the viral replication cycle at the level of adhesion, penetration, and possibly release. Other orthogonal approaches to further confirm viral replication, such as Western blot of viral proteins, electron microscopy, etc., could be useful. For further assessment of the function of HBGAs as attachment factors for hNoV, experimental blockage of the H type 1 antigen and analysis of its impact on viral replication *in vitro* would provide valuable additional insights. As unfiltered stool was used for the infection experiments, other interactions, enhancements, and blockages of hNoV infection could not be assessed due to the compositional complexity of untreated stool samples.

## Conclusion

The establishment of a Caco-2 cell line that is stably transfected with FUT2 and overexpresses the H type 1 antigen could be a step in the direction of establishing a productive cell culture system for hNoV by enhancing internalization of the virus. However, other receptors and cofactors also seem to play an important role. Furthermore, this study shows that viral release is restricted in this model, and this needs further examination.

## Data Availability

The datasets generated during and/or analysed during the current study are available from the corresponding author on reasonable request.

## Declarations

### Human and animal participants

This article does not contain any studies with human participants or animals and is in compliance with ethical standards for research.
